# A case-control study of bidi smoking and bronchogenic carcinoma

**DOI:** 10.4103/1817-1737.69116

**Published:** 2010

**Authors:** R. Prasad, R. C. Ahuja, S. Singhal, A. N. Srivastava, P. James, V. Kesarwani, D. Singh

**Affiliations:** *Department of Pulmonary Medicine, Chatrapati Sahuji Maharaj Medical University (Erstwhile K.G. Medical University), Lucknow, India*; 1 *Department of Clinical Epidemiology, Chatrapati Sahuji Maharaj Medical University (Erstwhile K.G. Medical University), Lucknow, India*; 2 *Department of Pathology, Era’s Lucknow Medical College, Lucknow, India*

**Keywords:** Bidi, cigarette, epidemiology, lung cancer, tobacco

## Abstract

**OBJECTIVE:**

To evaluate the risks imposed by tobacco smoking, in particular, bidi smoking, in the development of lung cancer.

**METHODS:**

Two hundred eighty-four histologically confirmed patients of bronchogenic carcinoma and 852 controls matched for age, sex, and socioeconomic status were interviewed according to a predesigned questionnaire. Effects of individual variables defining the various aspects of tobacco smoking, in particular, bidi smoking, were assessed using logistic regression models.

**RESULTS:**

81.3% cases of bronchogenic carcinoma were ever smokers as compared with 42.2% among controls. The odd ratios for ever smoking, bidi smoking, and cigarette smoking were 5.9 (confidence interval [CI] 4.3, 8.4), 6.1 (CI 4.3, 8.7), and 5.3 (CI 2.7, 10.4), respectively.

**CONCLUSION:**

Bidi smoking poses a very high risk for lung cancer even more than that of cigarette smoking.

″Bidis″ or ″beedis″ are small, hand-rolled unfiltered cigarettes that consist of tobacco flakes rolled in a tendu leaf (*Diospyros elanoxylon*) and tied with thread. They are also called ″beeris″ in countries, such as Bangladesh. The tobacco rolled in bidis is different from that used in cigarettes and is referred to as bidi tobacco.[1] In India, smoking accounts for majority of total tobacco consumption (72%), and among the total smoking habits, 73% is in the form of bidi and 27% is in the form of cigarette.[2] Roughly eight bidis are sold for every cigarette.[3]

In the last couple of years, reports of increase in the prevalence of bidi consumption have emerged from other countries in Asia, as well as other parts of the world, such as USA, France, Canada, and Australia.[4–9] Bidis are exported to around 30 countries from India and account for about 10% of the total tobacco export. During the eight years from 1995–1996 to 2003–2004, bidi exports have doubled.[10] Export of bidi to the USA is also on the rise due to a demand for flavored Indian bidis among the American youth.[11] They are also marketed internationally on the Internet. Internet sales of bidi pose several global challenges, including unrestricted sales to minors; lower prices through tax avoidance and smuggling; and unfettered advertising, marketing, and promotion.[12,13] The Internet as a marketing tool is largely unregulated and any existing regulation is difficult to enforce.

The leaf-wrapped appearance of bidis may also contribute to the perception among youth that bidis are ″safer, herbal″ cigarette.[14,15] The bidis are known as the ″poor man’s cigarettes,″ as they are smaller and cheaper than cigarettes and is perhaps the cheapest tobacco smoking product in the world.[16]

India accounts for more than 85% of the world’s bidi production.[17] But there are a few reports in which the association between bidi smoking and lung cancer has been specially analyzed.[18–23] The present case–control study was therefore undertaken to evaluate the risks imposed by tobacco smoking, in particular, bidi smoking, in the development of lung cancer.

## Methods

All the consecutive 284 newly diagnosed and histopathologically proven patients of bronchogenic carcinoma attending the Department of Pulmonary Medicine, Chatrapati Sahuji Maharaj Medical University (formerly King George’s Medical College), Lucknow, India, for treatment were included as cases. Of 284 patients, 133 (46.8%) had squamous cell, 67 (23.6%) small cell, 51 (18.0%) adenocarcinoma, 18 (6.3%) large cell, and 15 (5.3%) had other or mixed types of carcinomas. The patients were recruited between January 1992 and December 2001. Only those in whom the diagnosis of lung cancer was confirmed on cytologic or histologic examination of the material obtained from the primary site or a metastatic lymph node/pleural fluid with obvious primary lesion in the lungs detected radiologically, were included. Three hospital controls were selected for each patient from among the visitors and attendants of the patients. The controls were matched for age (±3 years), sex, and socioeconomic status. To exclude any respiratory disease, all the controls were subjected to clinical evaluation and chest radiography. Any control having history of past or recent onset cough, change in voice, hemoptysis, chest pain and presence of clubbing, or any significant lymph node on physical examination was excluded from the study.

Trained MD student (Tuberculosis and chest disease) interviewed the subjects in the hospital. The cases and controls were interviewed according to a pretested and validated questionnaire. The subjects were asked about identification particulars, socioeconomic parameters, and tobacco habits. Details of smoking habits were noted down carefully with regard to type (cigarette, bidi, chilam, which is a clay-pot containing a tobacco lit by fire; or hucca, which is a system where a chilam is attached to one end of a separate long wooden tube, while the other end of the tube is attached to a coconut-pot containing water and smoke thus passes over the water before it is inhaled), and amount and duration.

A smoking index was calculated as the average number of bidi or cigarettes consumed per day multiplied by the duration of smoking in years. The average number of cigarette or bidis smoked per day was calculated by summing up the smoking indices and dividing the whole by the duration of smoking in days. That is,

$$\frac{n_{1}d_{1}\;+\;n_{2}d_{2}\;+\;\cdot \cdot \cdot +\;n_{x}d_{x}}{D}$$

Where

*n* = Average number of sticks smoked per day during life time

*d* = Duration of smoking in days

*D* = Total duration of smoking (total of separate *d*’s)

The analysis was done for bidi smokers, cigarette smokers, as well as for overall smokers. Mixed smokers and Hukka smokers were excluded from the analysis. Nonsmokers were defined as individuals having exposure of <1 cigarette or bidi per day for less than one year.

### Statistical analysis

All analyses were performed using commercially available software (STATA version 6.0; Stata Corporation, 702 University Drive East, College Station, TX, USA). Statistical tests used were Chi-square test with fisher’s exact *P* value, and two-sample *t* test for parametric and Mann–Whitney *U* test for nonparametric and logistic regression.

## Results

One thousand one-hundred thirty-six participants were enrolled (284 cases and 852 controls) in the study, 94% of them were male and 84% were in between 41 and 70 years of age. The other sociodemographic characteristics (socioeconomic status, religion, and place of residence) were similar in both the groups.

The prevalence of overall smokers and bidi smokers in cases were approximately double than that of the control [Table 1]. It was also observed that various other types of smoking (cigarette, Hukka, or any combination) pattern were higher in cases than controls [Table 1].

**Table 1:** Prevalence of smoking habits

	Cases (n = 284)		Controls (n = 852)	
Smoking status	N	%	N	%
Nonsmokers	53	18.7	492	57.8
Bidi	195	68.7	297	34.8
Cigarette	19	6.7	33	3.9
Hukka	1	0.3	1	0.1
Mixed	16	5.6	29	3.4
Smokers	231	81.3	360	42.2

The number and duration of bidi and cigarette smoking was significantly higher in cases than that of control. The mean number of sticks smoked per day (bidi and cigarette both considered separately) was approximately 19 among cases and 11 among controls. The difference was statistically significant (bidi *P* < 0.0001 and cigarette *P* = 0.0056) [Table 2]. Duration of smoking was approximately 32 years for both cigarette and bidi smokers among cases which is higher as compared to controlm in which the duration was 28 years among bidi smokers and 25 years among cigarette smokers. This difference was also significantly higher than that of control (bidi *P* < 0.0001 and cigarette *P* = 0.0180) [Table 2]. Household smoke exposure does not have much significance as the number of females is less.

**Table 2:** Smoking habits—Number and duration

Smoking type	Cases	Controls	*P* value
No. of sticks (per day)			
Bidi	19.3 ± 9.6	10.9 ± 6.0	0.0000
Cigarette	19.6 ± 14.6	10.9 ± 8.5	0.0056
Duration (years)			
Bidi	32.8 ± 11.3	28.0 ± 11.6	0.0000
Cigarette	31.9 ± 8.9	25.0 ± 10.3	0.0180

To estimate the unadjusted odds ratio (OR) for developing bronchogenic carcinoma, overall smoking, only bidi smoking, only cigarette smoking, duration of bidi and cigarette smoking along with the number of times was considered in bivariate models. The odds of developing bronchogenic carcinoma among bidi smokers was 6 times more than that of nonsmokers (OR 6.1; 95% confidence interval [CI] 4.3, 8.7) [Table 3]. This was not much different from overall smokers (or 5.9; 95% CI 4.3, 8.4) and was little higher compared with the cigarette smoker group (OR 5.3; 95% CI 2.7, 10.4) [Table 3]. We also observed that the probability of developing bronchogenic carcinoma increases with the quantity and duration of bidi smoking.

**Table 3:** Smoking habits—Number and duration

Smoking status	Cases (n = 284)	Controls (n = 852)	OR	*P* value	95% CI
	Total (%)	Total (%)			
Non-smokers	53 (18.7)	492 (57.8)			
Total smokers	231(81.3)	360 (42.2)	5.9	0.0000	4.3, 8.4
Cigarette smokers	19 (6.7)	33 (3.9)	5.3	0.0000	2.7, 10.4
Bidi smokers	195 (68.7)	297 (34.8)	6.1	0.0000	4.3, 8.7
No. of bidis smoked/day					
1–10	39	178	2.0	0.0029	1.3, 3.3
11–20	92	101	8.5	0.0000	5.6, 12.9
>20	64	18	33.0	0.0000	17.6, 63.2
Years of smoking bidi					
1–20	36	88	3.8	0.0000	2.3, 6.3
21–39	131	177	6.9	0.0000	4.7, 10.1
≥40	28	32	8.1	0.0000	4.3, 15.1

CI = Confidence interval; OR = Odds ratio

Multiple logistic regression analysis reveals that after controlling for the duration of bidi smoking, the number of bidis smoked is strongly associated with the risk of bronchogenic carcinoma (OR 3.48; 95% CI 3.7, 4.5; *P* < 0.001) [Table 4], whereas after controlling for the number of bidis smoked per day, the duration of smoking was not significantly associated with the risk of bronchogenic carcinoma (OR 0.87; 95% CI 0.7, 1.1; *P* = 0.307) [Table 4].

**Table 4:** Logistic regression of number of bidis smoked and duration of smoking on bronchogenic carcinoma

Outcome: bronchogenic carcinoma	OR (95% CI)	*P* value
Number of bidis smoked/ day (0→ NS, 1→ 1–10, 2→ 11–20, 3→ >20)	3.48 (2.7, 4.5)	0.000
Duration (years) (0→ NS, 1→ 1–2–0, 2→ 21–40, 3→ >40)	0.87 (0.7, 1.1)	0.307
	*R*^2^ = 20.33	

CI = Confidence interval; OR = Odds ratio; NS = Not significant

## Discussion

Bidi smoking, having originated in India, is currently practiced all over the country and is the most popular form of tobacco use. In India, 8–10 times more bidis are smoked than cigarettes, a gross underestimation of the tobacco problem would occur by ignoring bidis. In our study, bidi smokers are 10 times more in number compared with cigarette smokers, which is in agreement with the national data, which indicate that our sample is representative of the whole population.

In our case–control study of 284 lung cancer cases and 852 controls, relative risk of bidi smoking was 6.1 (14.3, 8.7) and cigarette smoking was 5.3 (2.7, 10.4), which disproves the popular belief that bidi smoking is less harmful than cigarette smoking. The above result is in agreement with the previous studies, which also report the higher OR of bidi in comparison with cigarette. In a case–control study of 265 lung cancer cases and 525 hospital controls, the OR for bidi smokers was 5.76 (3.42–9.7), whereas that for cigarette smoking was 3.86 (2.11-7.06).[22] Another recent study from Chennai compared 778 lung cancer cases with 3430 controls.[23] The OR was 4.54 (2.96–6.95) and 6.45 (4.38–9.50) for more than 30 years of exclusive cigarette smoking and exclusive bidi smoking, respectively.[23] In a study from Mumbai, the OR for bidi and cigarette smoking was assessed from the analysis of 683 male lung cancer cases and 1279 male noncancer patients.[18] The OR of 3.38 for bidis was higher than the OR of 2.36 for cigarette smoking, compared with nonsmokers.[18] A previous hospital-based case–control study from Lucknow, comprising 52 cases of lung cancer with 156 healthy controls demonstrated that bidi smokers had an OR of 5.05 (2.21–11.7).[21]

Duration of bidi smoking is not significantly associated with the development of bronchogenic carcinoma, whereas the quantity of bidi smoked is. This harmful effect of bidi smoking may be due to the fact that mainstream smoke of bidi contains several toxic agents, such as hydrogen cyanide, carbon monoxide, ammonia, other volatile phenols, and carcinogenic hydrocarbons, such as benz(a)anthracene and benzopyrene.[24] Bidis typically deliver 3–5 times as much nicotine, tar, and carbon monoxide as conventional cigarettes.[25] It has been reported that bidis contain 1.5 times more carcinogenic hydrocarbons than American cigarettes.[26] The relatively low combustibility and nonporous nature of the tendu leaves require more frequent and deeper puffs by the smokers to keep bidi lit, and is therefore harder on the smoker’s lung than cigarette rolled in paper.[27] Bidi smokers were found to take almost five puffs per min compared with the cigarette smokers who smoked two puffs per min.[24] All these facts are responsible for greater deleterious effect of bidi.

It can be concluded that bidi smoking also poses a very high risk of lung cancer. Traditionally, tobacco control programs have focused on reducing cigarette consumption. Effective strategies are now needed to expand the focus of tobacco control programs to all types of tobacco use, including bidis.[28,29] Countries that adopted comprehensive tobacco control programs with a mix of interventions (including bans on tobacco advertising, controls on the use of tobacco in indoor locations, high taxes on tobacco products, and health education and smoking cessation programs) have had considerable success in decreasing the prevalence of cigarette smoking.[30] A similar policy framework with a mix of interventions have to be implemented to control bidi use in India and other Southeast Asian countries where bidi use is highly prevalent, as well as in countries, such as USA where the bidi market is relatively new and expanding.

Limitation of our study is that recall bias may be present in the cases and controls about their smoking habits.

## References

[1] Gupta PC, Reddy KS (2004). Report on tobacco control in India.

[2] Chaudhry K, Rath GK Multisectoral and intersectoral approach to national tobacco control. Paper commissioned by the World Health Organization on the occasion of the WHO International Conference on Global Tobacco Control Law: Towards a WHO Framework Convention on Tobacco Control. 2000 Jan 7-9, New Delhi, India.

[3] Srivastava A The role and responsibility of media in global tobacco control. Paper commissioned by the World Health Organization on the occasion of the WHO International Conference on Global Tobacco Control Law: Towards a WHO Framework Convention on Tobacco Control. 2000 Jan 7-9, New Delhi, India.

[4] Centers for Disease Control and Prevention (CDC) (1999). Bidi use among urban youth: Massachusetts, March-April 1999. MMWR Morb Mortal Wkly Rep.

[5] Healton C, Messeri P, Reynolds J, Wolfe C, Stokes C, Ross J (2000). Tobacco use among middle and high school students: United States 1999. MMWR Morb Mortal Wkly Rep.

[6] Indian hand-rolled cigarettes outpacing Sri Lankan brands. Hoover’s online. [last updated on 2001 Aug 31].

[7] Ansari KM French women blow hot with Indian bidi. Hindustan Times. [last updated on 2003 Nov 10].

[8] Barbara McLintock/The Province. Liberals move to ban bidis.

[9] Australian customs seize smuggled cigarettes from India. Wall Street Journal [last updated on 2001 Jul 17].

[10] (2004). Tobacco Board India. Export Reports. Exports of Tobacco Products from 1998-99 to 2003-2004. Guntur (Andhra Pradesh, India): Tobacco Board: Ministry of Commerce, Government of India.

[11] (1999). Tobacco Institute of India. Ban on import of Indian bidis.

[12] Cohen J, Sarabia V, Ashley M (2001). Tobacco commerce on the internet: A threat to comprehensive tobacco control. Tob Control.

[13] Milo N (2001). The internet, public health and the globalization of just about everything. J Epidemiol Community Health.

[14] Soldz S, Dorsey E (2005). Youth attitudes and beliefs toward alternative tobacco products: Cigars, bidis and kreteks. Health Educ Behav.

[15] Yen KL, Hechavarria E, Bostwick SB (2000). Bidi cigarettes: An emerging threat to adolescent health. Arch Pediatr Adolesc Med.

[16] Gupta PC, Asma S (2008). Bidi smoking and public health.

[17] Chaman Bidi Export: Amroha (Distt. Moradabad, U.P. India): Hashmi International Export. c2003-2004. http://www.tajbidi.comabout_bidi.htm.

[18] Notani PN, Rao DN, Sirsat MV, Sanghvi LD (1977). A study of lung cancer in relation to bidi smoking in different religious communities in Bombay. Indian J Cancer.

[19] Jindal SK, Malik SK, Dhand R, Gujral JS, Malik AK, Datta BN (1982). Bronchogenic carcinoma in Northern India. Thorax.

[20] Malhotra V, Malik R, Beohar PC, Gondal R, Khanna SK, Narayanan PS (1986). Tumours of the lung: A histopathological study. Indian J Chest Dis Allied Sci.

[21] Prasad R, Tandon S, Kumar S, Pant MC, Sinha KN, Mukerji PK (1998). A case control study on tobacco smoking and lung cancer. Lung India.

[22] Gupta D, Boffetta P, Gaborieau V, Jindal SK (2001). Risk factors of lung cancer in Chandigarh, India. Indian J Med Res.

[23] Gajalakshmi V, Hung RJ, Mathew A, Varghese C, Brennan PK (2003). Tobacco smoking and chewing, alcohol drinking and lung cancer risk among men in southern India. Int J Cancer.

[24] Jayant K, Pakhale SS, Sanghavi LD, Notani P (1989). Toxic constituents in bidi smoke. Tobacco and Health: The Indian Scene.

[25] Rickert WS (1999). Determination of yields of ″tar″, nicotine and carbon monoxide from Bidi, cigarettes: Final report.

[26] Hoffman D, Sanghvi LD, Wynder EL (1974). Comparative chemical analysis of Indian bidi and American cigarette smoke. Int J Cancer.

[27] Bhonsle RD, Murti PR, Gupta PC, Gupta PC, Hammer JE, Murti PR (1992). Tobacco habit in India. Control of tobacco related cancers and other disease. Proceedings of an International Symposium; 1990 Jan 15-19; Bombay, India.

[28] Global Youth Tobacco Survey Collaborative Group (2003). Differences in worldwide tobacco use by gender: Findings from the Global Youth Tobacco Survey. J Sch Health.

[29] Global Youth Tobacco Survey Collaborative Group (2002). Tobacco use among youth: A cross country comparison. Tob Control.

[30] (2002). World Health Organization. Reducing risks, promoting healthy life. The World Health Report, 2002.

